# Using Internet Search Engines to Obtain Medical Information: A Comparative Study

**DOI:** 10.2196/jmir.1943

**Published:** 2012-05-16

**Authors:** Liupu Wang, Juexin Wang, Michael Wang, Yong Li, Yanchun Liang, Dong Xu

**Affiliations:** ^1^Key Laboratory of Symbol Computation and Knowledge Engineering of Ministry of EducationCollege of Computer Science and TechnologyJilin UniversityChangchunChina; ^2^School of MathematicsJilin UniversityChangchunChina; ^3^Department of Computer Science and Christopher S. Bond Life Sciences CenterUniversity of MissouriColumbia, MOUnited States; ^4^Department of Pathology and Anatomical Sciences and Ellis Fischel Cancer CenterUniversity of MissouriColumbia, MOUnited States; ^5^Department of Computer ScienceUniversity of MissouriColumbia, MOUnited States

**Keywords:** Internet search, page rank, Google, Yahoo!, Bing, Ask.com, medical information, health information seeking, breast cancer, SNOMED CT, user experience evaluation, usability testing, hallway testing, software engineering

## Abstract

**Background:**

The Internet has become one of the most important means to obtain health and medical information. It is often the first step in checking for basic information about a disease and its treatment. The search results are often useful to general users. Various search engines such as Google, Yahoo!, Bing, and Ask.com can play an important role in obtaining medical information for both medical professionals and lay people. However, the usability and effectiveness of various search engines for medical information have not been comprehensively compared and evaluated.

**Objective:**

To compare major Internet search engines in their usability of obtaining medical and health information.

**Methods:**

We applied usability testing as a software engineering technique and a standard industry practice to compare the four major search engines (Google, Yahoo!, Bing, and Ask.com) in obtaining health and medical information. For this purpose, we searched the keyword *breast cancer *in Google, Yahoo!, Bing, and Ask.com and saved the results of the top 200 links from each search engine. We combined nonredundant links from the four search engines and gave them to volunteer users in an alphabetical order. The volunteer users evaluated the websites and scored each website from 0 to 10 (lowest to highest) based on the usefulness of the content relevant to breast cancer. A medical expert identified six well-known websites related to breast cancer in advance as standards. We also used five keywords associated with breast cancer defined in the latest release of Systematized Nomenclature of Medicine-Clinical Terms (SNOMED CT) and analyzed their occurrence in the websites.

**Results:**

Each search engine provided rich information related to breast cancer in the search results. All six standard websites were among the top 30 in search results of all four search engines. Google had the best search validity (in terms of whether a website could be opened), followed by Bing, Ask.com, and Yahoo!. The search results highly overlapped between the search engines, and the overlap between any two search engines was about half or more. On the other hand, each search engine emphasized various types of content differently. In terms of user satisfaction analysis, volunteer users scored Bing the highest for its usefulness, followed by Yahoo!, Google, and Ask.com.

**Conclusions:**

Google, Yahoo!, Bing, and Ask.com are by and large effective search engines for helping lay users get health and medical information. Nevertheless, the current ranking methods have some pitfalls and there is room for improvement to help users get more accurate and useful information. We suggest that search engine users explore multiple search engines to search different types of health information and medical knowledge for their own needs and get a professional consultation if necessary.

## Introduction

The Internet is becoming one of the most important sources to obtain medical and health information for the general public [1]. The Pew Internet & American Life Project reported [2] that about 80% of Internet users look for medical or heath-related information through the Internet. It has been found that the most common health and medical topics searched on the Internet were a specific disease or medical problem, certain medical treatment, diet, nutrition and vitamins, and exercise or fitness [2], as well as prescription drugs [3]. To help the public obtain accurate and useful medical information, the US National Library of Medicine developed an official website (MedlinePlus; www.nlm.nih.gov/medlineplus/) to provide consumers with access to current full-text publications in health and medicine [4]. Volunteers from universities, hospitals, and research institutions summarize their work in the health sections of Wikipedia (www.wikipedia.org). The editors have invited the medical community to edit and update the content in Wikipedia to provide reliable and understandable health information [5]. It was suggested that such a World Wide Web-based supporting system would become an important professional tool for future medicine [6]. Researchers also explored the feasibility of using online medical information as a diagnostic aid [7]. An online patient recruitment company studied patients’ search queries and questions for clinical trial information in order to improve the clinical trial recruitment process [8]. Thus, the Internet has become an indispensable source for the public, patients, and health care professionals to obtain information about health, diseases, and medical treatment.

Various investigations have been conducted for improving search methods to help users obtain accurate and useful information [9]. Some studies have improved the search results or efficiency by analyzing the purpose of the Internet search [10]. In particular, researchers have classified and analyzed the information needs of users to improve search engines [11]. A medicine-related information search is different from other information searches, since users often use medical terminology, disease knowledge, treatment options, and so on. Hence, some studies targeted the characteristics and motivations of health information seekers and the usage of online medical information [12,13]. As early as 1997, Bonati’s group studied the reliability of health care information from two major search engines, Yahoo! and Excite. They found that only a few websites provided the comprehensive and accurate information sought due to lack of mature techniques of most search engines used for public health care information [14]. In 1999, researchers compared the accuracy and reliability of search results from five search engines: AltaVista, Excite, Hotbot, Infoseek, and Lycos [15]. A similar study compared two major search engines, Alltheweb and Excite [16]. Their results demonstrated that the use of the general search engines for health care information, as well as the use of specialized medical and health websites, had dramatically increased. Another area of study is the user experience in health care information search [17]. In 2005, Forrester Research, Inc. conducted a Web survey to assess search engines by evaluating AOL, Google, MSN, and Yahoo! according to 11 parameters in user experience [18]. However, it is difficult to obtain a comprehensive evaluation for the search engines based only on these scores without a medical professional’s input.

While several major search engines are available, most users limit their Internet search to one search engine. This search habit raises several questions regarding the search for medical information. Does a single search engine provide reliable medical information? Are search results from different engines similar when using multiple search engines? Does searching multiple search engines for the same queries add value? Is any search engine significantly better than the others? These questions become more relevant as the market shares of Internet search engines are evolving. A report from comScore, a global company measuring the digital world and the preferred source of digital marketing intelligence, showed that in September 2009, the top five search engines on the market were Google, Yahoo!, Microsoft Sites, Ask Network, and AOL LLC [19]. According to comScore, Google has about two-thirds of the US market share in Internet searching, and its share is increasing. Then a natural question is whether Google is enough for medical information searching without needing to check other search engines.

Among all the medical search queries on the Internet, cancer-related information is one of the most popular topics. In particular, breast cancer, as the most common cancer and the second-leading cause of cancer deaths among American women, draws much of the public’s attention, especially on the Internet [20]. In a previous study [21], we recruited some Internet users and a medical professional, and asked them to score the content of webpages resulting from Google searches to reflect their user experience quantitatively from a software engineering perspective. This pilot study showed that usability testing is very useful for evaluating Internet searches in obtaining medical information. It also showed that the specificity of Google in searching for medical information is often satisfactory. However, the study targeted only one search engine, Google. The current study extended our early work by adding Yahoo!, Bing, and Ask.com, which allowed us to conduct a comparative study. Among the four search engines, Bing is a new one built in 2009 by Microsoft. We still chose *breast cancer *as a keyword and Systematized Nomenclature of Medicine-Clinical Terms (SNOMED CT) [22,23] as a standard reference in our validation process. We used Java Vector data structure to read and record the ranking, website name, and URL of webpages in the search result list.

## Methods

### Design

We used the following protocol to conduct our research. First, search *breast cancer *in Google, Yahoo!, Bing, and Ask.com and save the top 200 websites shown in each search, including website name, URL, and ranking of each result in every group. Second, apply the Google PageRank tool to check and record the Google PageRank for each search result in every group. Third, compare website links to check overlapping of search results from the same search engine and between the different search engines. Fourth, define standards of the most helpful and commonly used websites for breast cancer by a breast cancer research expert, and have eight volunteers study the standard websites so that they would know what kinds of websites are informative. Fifth, ask the eight volunteers to score each of the combined, nonredundant websites in alphabetical order, scoring from 0 (a useless website) to 10 (the most helpful website). We would then collect the data from volunteers and rerank the websites from high to low in every group (corresponding to each search engine). Sixth, classify each search result according to its webpage content, with four types of content defined. Seventh, use Java.net to retrieve the text of all nonredundant websites, and use the subpages to study the pattern of usage of the keyword *breast cancer *and its related SNOMED CT terms. Eighth, analyze the data and compare the effectiveness of Google, Yahoo!, Bing, and Ask.com in obtaining medical and health information.

### Collection of Search Results

On October 27, 2009, we used four search engines—Google, Yahoo!, Bing, and Ask.com—to search *breast cancer *with the default search parameters. A Java application programming interface named JExcelApi [24] was used to record related data into an Excel (Microsoft Corporation, Redmond, WA, USA) file automatically. We obtained the search results using the Windows 7 Professional Service Pack 1 (Microsoft Corporation) operating system in English. All the searches and their evaluations were carried out on machines physically located in Columbia, Missouri, USA.

We obtained a total of 798 search results in the following four groups. Google (www.google.com) obtained 40,800,000 search results, and the response time was 0.13 seconds. We chose the top 200 websites from these results to compose the Google sample group in this study. Yahoo! (www.search.yahoo.com) obtained 262,000,000 search results. We chose the top 200 websites from these results as the Yahoo! sample group. Bing (www.bing.com) obtained 74,500,000 search results. We chose the top 200 websites from these results as the Bing sample group. Ask.com (www.ask.com) had 9,080,000 search results. We chose all 198 websites available to users for the Ask.com sample group. Ask.com is a metasearch engine, which aggregates and selects the results from several other search engines into a single list.

**Figure 1:**
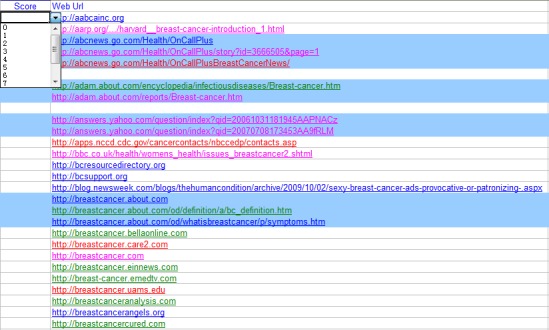
Sample of score list used by volunteers.

Then we combined the nonredundant links and provided them to volunteer users in alphabetical order, as Figure 1 shows. Redundancy is defined by websites having the same URL address. Sometimes, different URLs or even domain names have identical or similar content. However, such cases are relatively rare and it is very time consuming to manually label them. Hence, we did not consider this issue in this study. We used alphabetical order so as to avoid the bias from ranked order, as volunteers may unconsciously have followed the search engines’ ranking of the search results. Using nonredundant links reduced the volunteers’ workload, from 798 search results to 592 Web links. At the same time, we also grouped the sample search results together if they share the same domain name for the user’s convenience.

### PageRank Value of Search Results

The Google search engine has a prominent status and a large market share in Internet search. The PageRank algorithm is one of Google’s core technologies. The PageRank value is a parameter to evaluate the importance of a website, ranging from 0 to 10 [25]. Many software tools for research or business use the Google PageRank technology. We used the PageRank Checker Tool [26] and the Google PageRank Checker [27] to evaluate and record the Google PageRank value for each search result in every group.

### Scoring Criteria

The volunteers evaluated each website from 0 to 10 based on its usefulness concerning breast cancer, where a rating of 10 indicated the most helpful website. To orientate the volunteers, Dr Michael Wang, a physician and a breast cancer researcher, defined gold standard websites that provide the most useful information about breast cancer to the public. These websites usually have all the basic information about breast cancer, such as understanding breast cancer, symptoms, and diagnosis. It is convenient for the public to obtain the information they need rapidly from these websites. He identified six gold standard websites: standard No. 1, from the US National Cancer Institute; standard No. 2, from the American Cancer Society; standard No. 3, from the Mayo Clinic; standard No. 4, from MedicineNet; standard No. 5, Wikipedia; and standard No. 6, from Susan G. Komen for the Cure.

### Evaluation of Websites by Volunteers

We provided a list of nonredundant search results to eight nonphysician volunteers. Each volunteer had at least 5 years’ experience of using the Internet. The volunteers first studied the six standard websites so that they knew what they could expect from high-quality websites, annotated by the expert. However, they did not have to give these sites high scores if they did not regard them as helpful. Then they read the sample websites individually and scored them based on the standard and their own experience. The eight volunteers in this study were from the University of Missouri-Columbia: volunteer 1 was a 30-year-old female PhD student with biochemistry background; volunteer 2 was a 36-year-old male Associate Professor with a PhD in computer science; volunteer 3 was a 31-year-old male PhD student in computer science; volunteer 4 was a 29-year-old male PhD student in computer science; volunteer 5 was a 23-year-old female undergraduate student in biochemistry; volunteer 6 was a 27-year-old male graduate student in chemistry; volunteer 7 was a 25-year-old male graduate student in economics; and volunteer 8 was a 22-year-old female undergraduate student in biological science.

These volunteers were frequent Internet users but did not have any background in medical science. The method of choosing and inviting testing users is different from the regular way of randomly choosing volunteers in hallway testing [28]. A typical hallway testing limits the workload of each volunteer to 10 minutes with a questionnaire containing no more than 50 questions. In this study each volunteer evaluated 592 sample websites. Browsing and scoring all of them took about 10 hours per volunteer on average. Given the heavy workload, we made sure that each volunteer was committed to participating in the study. During the study, we advised each volunteer to do the study whenever he or she could find some time but to finish the evaluation as soon as possible to reduce the negative effect caused by the expiration of some Web links. It took from 3 to 17 days to finish evaluating all the webpages of the search results. Since many of the search results contained medical terminologies and related knowledge, we only selected volunteers with a higher-education background so that they could understand the content. The volunteers’ experience in Internet use ensured that they could conduct the study smoothly [29]. The users’ age and gender were diverse, following early suggestions [30,31]. Overall we attempted a representative study given the availability of volunteers.

### Categorization of Search Results

We studied the search results by classifying their webpage content, as different categories of content may have an impact on users’ scores. We manually classified 798 search results into the following four types: type 1, websites for basic breast cancer knowledge targeting the general population such as news sites, Web tribunes, personal websites, and blogs; type 2, nonprofit organization websites for breast cancer patients and their families including websites of breast cancer societies and foundations; type 3, corporate websites for consumers including advertisement websites for medicine, devices, products, and so on; and type 4, websites for breast cancer professionals and researchers such as websites from universities, research institutions, hospitals, and government.

### Ranking Based on Selected Keywords

We provided a reference ranking based on the occurrence of selected keywords. We used Java.net to obtain the text content automatically from all websites. All the text in a website in the search result formed the main page text corpus. All the subpages of this website combined with corpus A formed the main and subpages text corpus. Based on the description of breast cancer in SNOMED CT, we selected four more keywords: *malignant tumor*, *neoplasm*, *sarcoma*, and *carcinoma*. We reranked the websites in each group based on the frequency of these keywords in the text corpus according to the following rules: (1) the website with the most types of keywords is ranked on top, (2) if 1 is inconclusive, the one with greater occurrence of *breast cancer *is on top, (3) if 2 is inconclusive, the one with more hits of the other three key words is on top, and (4) if 3 is inconclusive, the one with higher ranking in the original search result list is on top.

## Results

### Validity of Search Results

Search validity is defined according to whether a user can successfully open the URL of a search result. Our analysis showed that the validities of the search results from the four search engines were significantly different. The validity of Google search results was 100% (200/200), while Yahoo! had a validity of 92.5% (185/200) with 15 invalid results (websites 21, 53, 75, 85, 118, 119, 120, 126, 129, 140, 147, 149, 162, 171, and 176 in Multimedia Appendix 1, Table 1), Bing had a validity of 98.5% (197/200) with three invalid results (74, 148, and 149), and Ask.com had a validity of 98.9% (196/198) with two invalid results (12 and 83). This indicates that Google probably updates its database of search results more frequently than other search engines. We did not use the cached pages of the invalid sites for any further study. The cached pages are typically incomplete and they are often ignored by users. Hence, they are not suitable for usability studies.

### PageRank Value of Search Results

**Figure 2:**
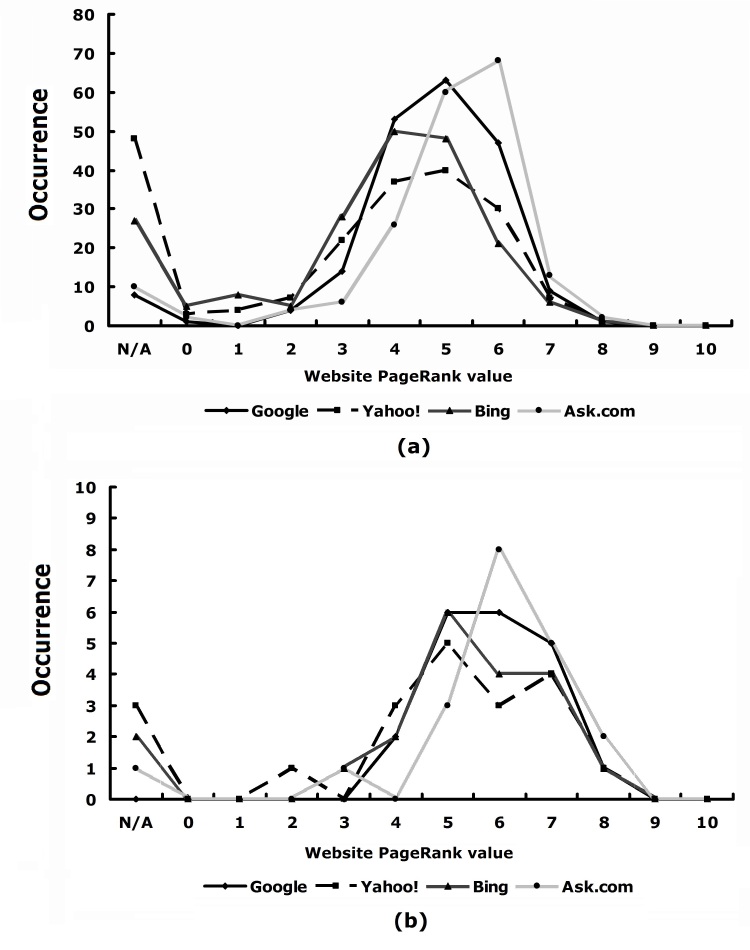
Distribution of PageRank values of search results from Google, Yahoo!, Bing, and Ask.com. (a) All search results, (b) top 20 search results. N/A = not available.

Figure 2a shows the PageRank distribution of search results in each of the four groups. The distributions of the four groups were similar, although Ask.com skewed more toward higher PageRank values than the other three. It is likely that Ask.com as a metasearch engine selects the results from other search engines by considering a factor similar to PageRank. Since many Internet search users look at only the top 10 or 20 hits, we also compared the search engines using the top 20 search results. Figure 2b. It shows that the features are almost the same as those in the top 200 hits.

### Ranking of Six Standard Websites

For the six standard websites, four search engine hits contained the exact URLs for five of them. The other one (standard No. 2, http://www.cancer.org/docroot/home/index.asp) was identified by two search engines (Bing and Ask.com), and its subpage (http://www.cancer.org/docroot/cri/cri_2x.asp?sitearea=lrn&dt=5) appeared in the Google and Yahoo! search results as Google No. 16 and Yahoo! No. 1, respectively. Counting this subpage, all six standard websites appeared on the top 30 of each search engine’s results, except for one (standard No. 3 ranked 33 by Bing), as Table 1 shows.

**Table 1:** Ranking of six standard websites.

Website No.	Website name	Website URL	Search engine ranking			
			Google	Yahoo!	Bing	Ask.com
1	National Cancer Institute: Breast cancer	http://www.cancer.gov/cancertopics/types/breast (not archived)	4	9	3	7
2	American Cancer Society: Information and Resources for Cancer	http://www.cancer.org/docroot/home/index.asp (not archived)	16	1	5	13
3	MayoClinic: Breast cancer	http://www.mayoclinic.com/health/breast-cancer/DS00328 (http://www.webcitation.org/67hgmdCNF)	9	17	33	22
4	MedicineNet.com: Breast cancer	http://www.medicinenet.com/breast_cancer/article.htm (http://www.webcitation.org/67hgtCTA8)	8	26	4	9
5	Wikipedia: Breast cancer	http://en.wikipedia.org/wiki/Breast_cancer (http://www.webcitation.org/67hh3W7P8)	13	23	2	5
6	Susan G. Komen Breast Cancer Foundation (http://www.webcitation.org/67hh7GieT)	http://ww5.komen.org/	3	10	8	6

### Overlap Between Search Results

We found that only 397 of 798 (49.8%) search results were nonredundant, with 401 results having a duplicate URL. In addition, 466 of 798 (58.4%) sample results had redundant domain names. As Table 2 shows, within each group Google search results had no redundant URLs, but 18 of the 200 websites (9%) had domain names redundant with other search results in the same group. This is much less than in the other three groups, where Yahoo!, Ask.com, and Bing had domain redundancy of 12% (24/200), 16% (31/198), and 16% (32/200), respectively. Too much redundancy may lead to poor user experience. Thus, it is an important factor in user experience evaluation.

**Table 2:** Redundancy of search results.

Search engine	Google (n = 200)		Yahoo! (n = 200)		Bing (n = 200)		Ask.com (n = 198)	
	URL redundancy	Domain redundancy	URL redundancy	Domain redundancy	URL redundancy	Domain redundancy	URL redundancy	Domain redundancy
Google	0	18	61	75	49	67	67	79
Yahoo!	61	75	2	24	60	76	42	52
Bing	49	67	60	76	2	32	38	61
Ask.com	67	79	42	52	38	61	13	31

In Table 2, as an example, Google and Yahoo! shared 61 results with the same URLs and 75 results with the same domain names.

### Categorization Distribution of Search Results

**Figure 3:**
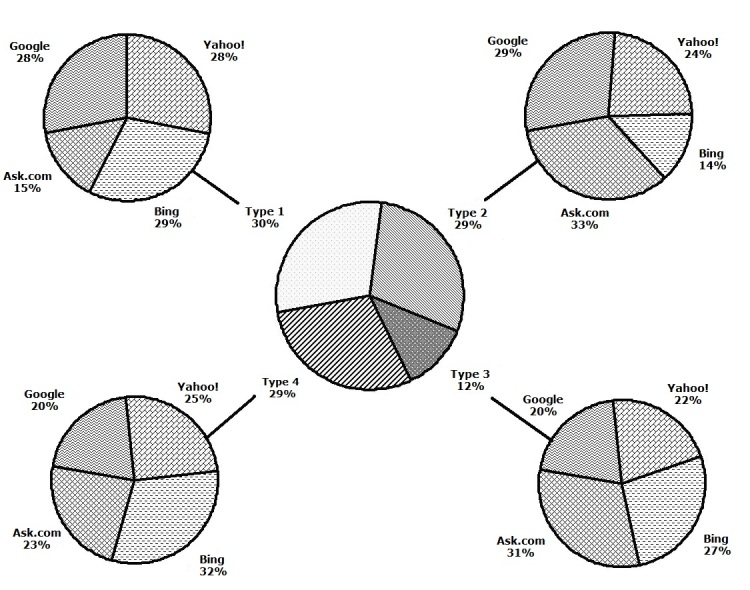
Distributions of the four webpage types among all search results and in the four search engines.

**Figure 4:**
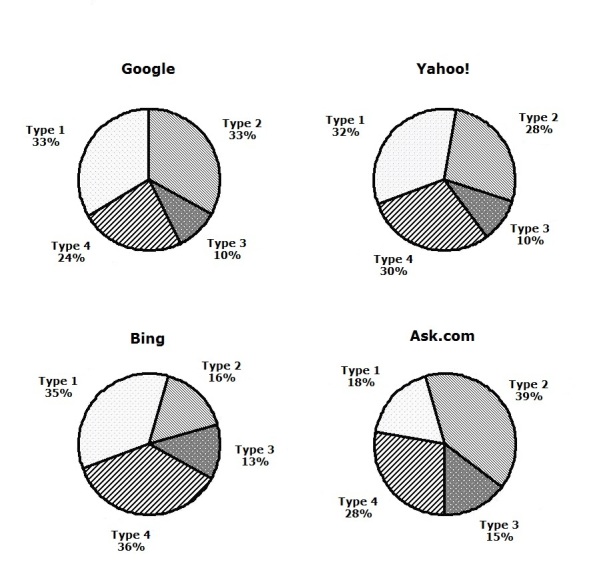
Distribution of the four webpage types in each search results group (by search engine).

Figure 3 and Figure 4 show that the four search engines differed in their distributions of search results in terms of content types. To review, the four types of websites are (1) basic knowledge websites targeting the general population, (2) nonprofit organization websites, (3) corporation websites, and (4) websites for professionals and researchers. Overall, most results fell into type 1, type 2, and type 4, with proportions all around 30%. The distributions of types 1–4 were 29.7% (237/798), 29.2% (233/798), 12% (93/798), and 29.4% (235/798), respectively. Google mostly covered type 1 and type 2, with 33% (66/200) and 34% (67/200) of all Google search results, respectively. Yahoo! had overrepresentation in type 1 (33% (66/200) among all Yahoo! search results). Bing emphasized type 1 and type 4 (35% (70/200) and 37% (73/200) among all Bing search results, respectively). Ask.com focused on type 2 (40% (79/198) among all Ask.com search results).

### PageRank Values of Four Webpage Types

**Figure 5:**
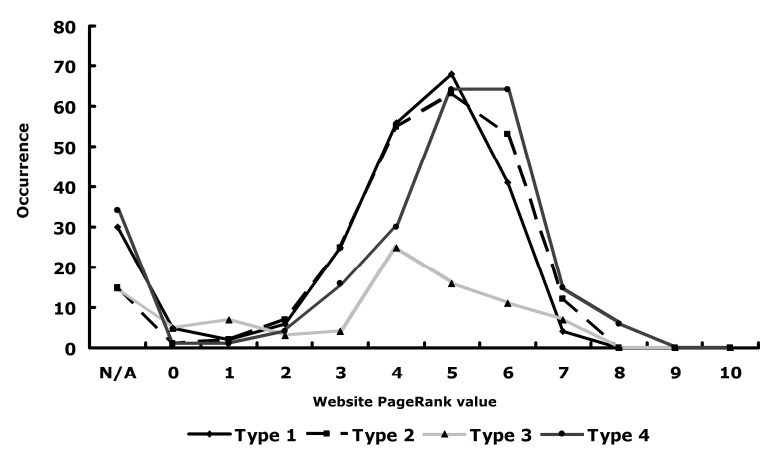
PageRank value of the four webpage types. N/A = not available.

Figure 5 shows the distribution of the PageRank value among the different types of websites. The average PageRank values of the four types are 4.48 for type 1, 4.72 for type 2, 4.03 for type 3, and 5.14 for type 4. It is understandable that type 4 has the highest PageRank value, as these websites contain authoritative content from breast cancer professionals and researchers. Because there are more personal websites (instead of institutional websites) in type 1, the popularity of these websites tends to be lower, and hence type-1 websites overall have lower PageRank values. Since type 3 (corporate websites) is mainly related to advertisement, users might tend to avoid these websites, and hence they also have lower PageRank values overall. Many of the type-2 (nonprofit) websites with high PageRank values belong to the same group—for example, the American charity website CharityUSA.com.

### Volunteers’ Scores for Standard Websites

While the volunteers were given the standard websites, they did not have to give the sites a score of 10. While some sites had high scores (eg, the volunteers gave standard No. 5 an average score of 9.88), it is interesting that volunteers often gave some sites relatively low scores, as Table 3 shows. In particular, standard No. 2 (http://www.cancer.org/docroot/home/index.asp) had an average score of only 6.50; this website was the only one that was not identified by all 4 search engines. This shows the due diligence of the volunteers.

**Table 3:** Volunteers’ scores for the six standard websites.

Website No.	Volunteer No.								Average score
	1	2	3	4	5	6	7	8	
1	10	9	10	9	8	8	10	6	8.75
2	6	8	8	6	5	7	7	5	6.50
3	10	8	10	9	9	9	10	5	8.75
4	10	10	10	9	9	8	9	8	9.13
5	10	10	10	10	10	10	10	9	9.88
6	10	9	10	8	10	7	10	6	8.75

### Scores of Search Results From Different Search Engines

**Figure 6:**
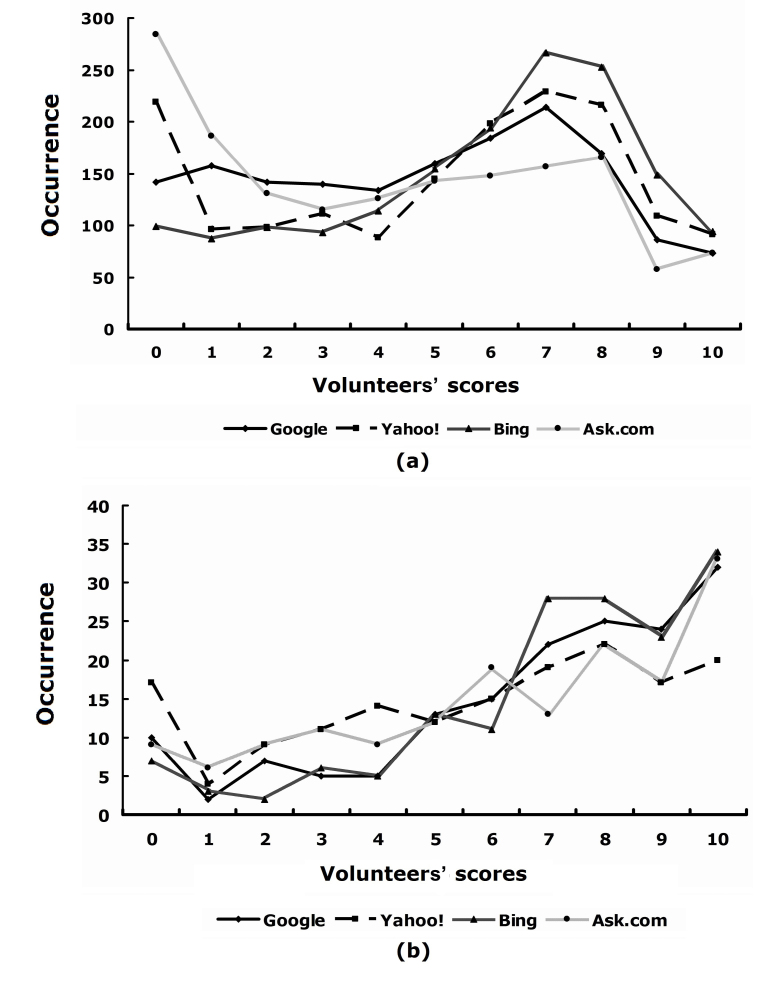
Distribution of websites with different scores in each search group (all eight volunteers’ scores aggregated). (a) All search results, (b) top 20 search results.

Figure 6a shows the score distribution in each search group. Distribution patterns were similar in Google and Bing, and in Yahoo! and Ask.com. Bing had the highest average score at 5.70, with more high scores from 7 to 10, while Yahoo!, Google, and Ask.com had average scores of 5.07, 4.78, and 4.10, respectively. Figure 6b shows the score distribution for top 20 search results in each search group. Bing again had the highest average score at 7.14, while Google, Ask.com, and Yahoo! had average scores of 6.85, 6.36, and 5.77, respectively.

**Figure 7:**
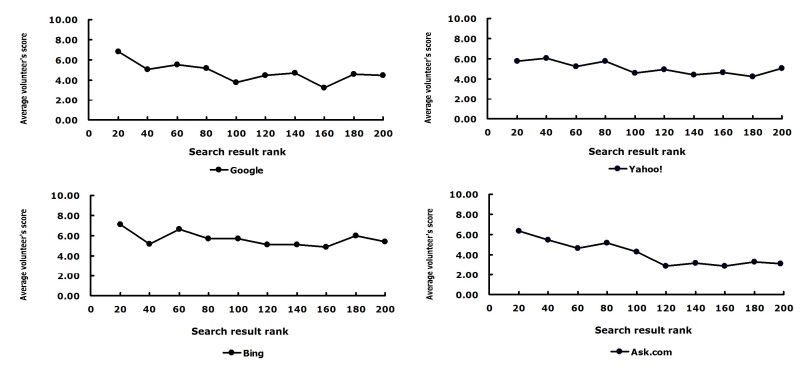
Volunteer’s score versus search result rank (with a window size of 20) in each search group.

Figure 7 shows the average score versus ranking with a window size of 20. Table 4 provides some additional assessment. The general trend of the curves in Figure 7 is as expected—that is, the higher the ranking, the higher the volunteer’s score. In terms of the correlation between the scores and the ranking by the search engine, the best search engine was Ask.com, followed by Google, Bing, and Yahoo!. A minor issue in this comparison is that each search engine had some redundant search results or domain names. Given that the redundancy was not very high, we ignored the issue for the comparison between different search engines.

**Table 4:** Performance (average score) of the four search groups.

Performance measure	Google	Yahoo!	Bing	Ask.com
Top 10 websites	7.34	5.46	7.70	6.79
Top 20 websites	6.85	5.77	7.14	6.36
Top 50 websites	5.96	5.85	6.19	5.65
Top 100 websites	5.28	5.49	6.08	5.19
Total	4.78	5.07	5.70	4.14
Pearson correlation coefficient^a^	–.3036	–.1937	–.1964	–.5099
Spearman rank correlation^a^	–.3062	–.2051	–.2281	–.4725

^a ^Pearson correlation coefficient and Spearman rank correlation coefficient are between volunteer’s score and ranking by the search engine. The larger the absolute value, the greater the correlation in this case.

### Difference Between Evaluations of Various Users

**Figure 8:**
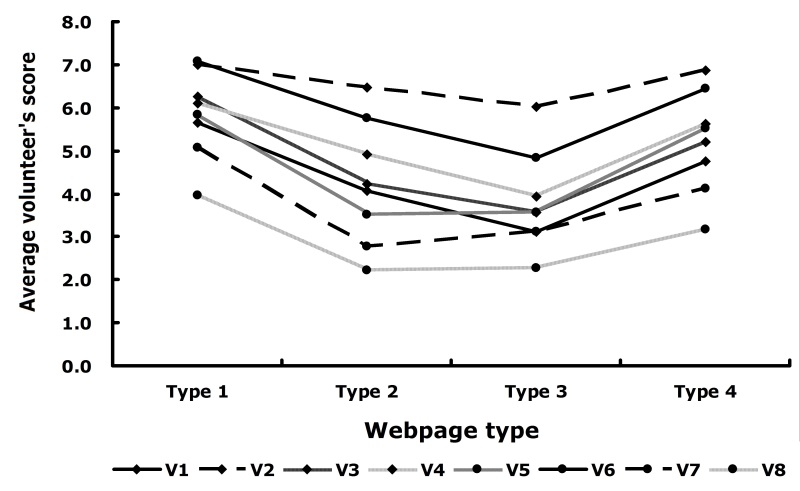
Average volunteer's scores for the four webpage types. V1-8 = volunteers 1 to 8.

Volunteers had different user experiences and different schemes for grading webpages. Figure 8 plots each volunteer’s average scores for the four website types. Volunteer 1, volunteer 2, volunteer 3, volunteer 4, and volunteer 6 had similar tendencies, while volunteer 5, volunteer 7, and volunteer 8 had similar tendencies. Interestingly, the latter three volunteers were the youngest in the pool, although the sample size is too small to draw a conclusion on the effect of age.

### Keyword Frequencies in Search Results

Table 5 shows the occurrence in the webpages of *breast cancer *and the four other related keywords defined by SNOMED CT (*malignant tumor*, *neoplasm*, *sarcoma*, and *carcinoma*). The number of hits for the keyword *breast cancer *is much larger than that of any of the other four. The keyword *breast cancer *occurred among 91.71% (22,301/24,317) of the main page text corpus, and 88.53% (261,067/294,875) of the main and subpages text corpus. *Carcinoma *was the second most-frequent keyword, with an occurrence of 6.08% (1479/24,317) in the main page text corpus and of 6.87% (20,251/294,875) in the main and subpages text corpus.

**Table 5:** Occurrence of keywords in the main page text corpus (MP) and in the main and subpage text corpus (MSP).

Search engine			Breast cancer	Malignant tumor	Neoplasm	Sarcoma	Carcinoma
Google							
	MP		4619	14	56	62	363
	MSP		54,511	61	793	570	2629
Yahoo!							
	MP		6423	28	77	45	399
	MSP		70,471	128	1457	1085	5104
Bing							
	MP		8171	23	80	80	548
	MSP		89,577	143	2231	5355	10,397
Ask.com							
	MP		3088	2	37	33	169
	MSP		46,508	23	730	981	2121
Total							
		MP	22,301	67	250	220	1479
		MSP	261,067	355	5211	7991	20,251
Percentage							
		MP	91.71%	0.3%	1.0%	0.90%	6.08%
		MSP	88.53%	0.12%	1.77%	2.71%	6.87%

**Figure 9:**
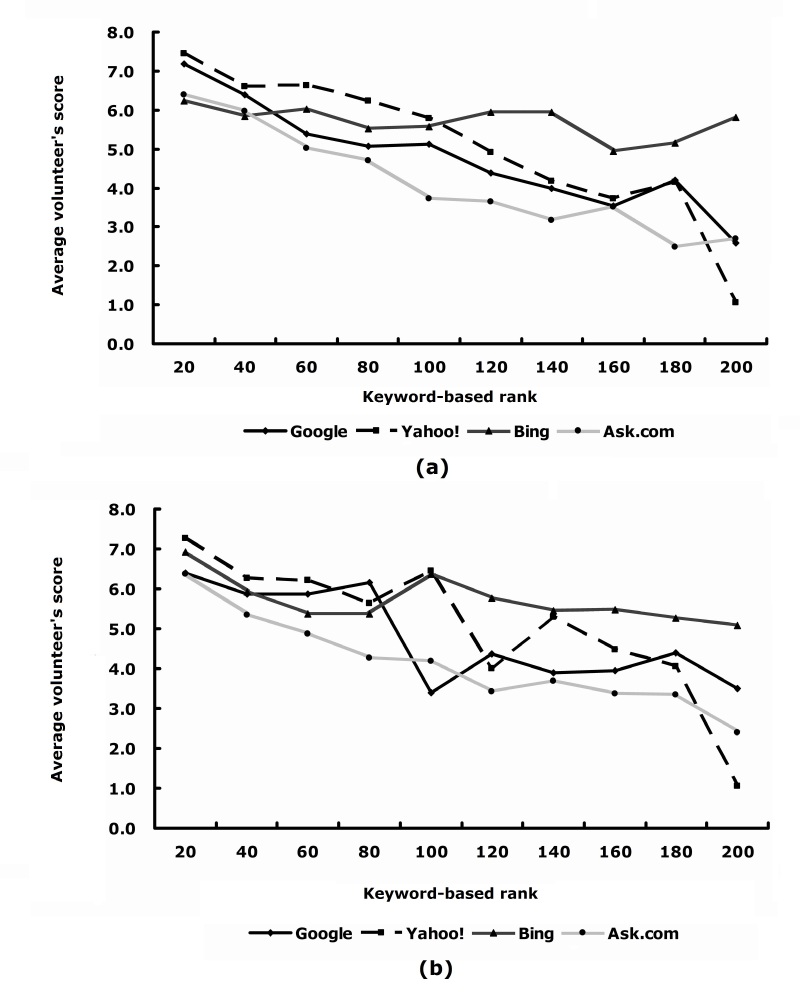
Average volunteer’s score versus keyword-based rank in (a) the main page text corpus and (b) the main and subpage text corpus.

Figure 9 shows the average scores versus keyword-based reranking (as described in the subsection “Ranking based on selected keywords”) with an interval of 20 search results (top 1–20, ranks 21–40, ranks 41–60, etc) for each search group. As the keyword-based ranking decreases, the average score also decreases, which indicates that the scores reflect an accurate representation of keywords.

## Discussion

In this study, we compared and evaluated four major search engines for medical information search using various assessment criteria. The search results were significantly different between any two search engines. Yahoo! had the lowest performance scores, which does not correspond to its second ranking in the market. A possible explanation of this discrepancy is that Yahoo! has been a major search engine for a long time and its other functions result in some good user experiences. Ask.com was not as good as Google and Bing. While Ask.com had a higher correlation between the scores and the ranking by the search engine than either Google or Bing, the volunteers scored its top hits lower, indicating a less useful status, as most users browse just the top 10 to 20 hits. Google and Bing each have some merits. Google search results had higher validity and less redundancy. On the other hand, volunteers regarded the top hits of Bing as being more useful.

By studying the distribution of four types of webpages, we found that the search engines had different priorities for various categories of search results as shown in Figure 3 and Figure 4. Statistical results show that the content and type of search results from the Google and Yahoo! search engines were diversified with balanced content, and with a relatively low amount of advertisement content. In contrast, Bing had unbalanced search results, covering more breast cancer knowledge for the general population and professionals but fewer nonprofit organization websites and corporate websites for consumers. Bing’s search results contained more advertisements than Google and Yahoo!. Ask.com also had unbalanced search results, emphasizing nonprofit organization websites for breast cancer patients and their families. Ask.com’s search results had excessive advertisements, and its ability to control the advertisement information was the worst among the four engines, which might be related to the features of a metasearch engine [32].

Volunteers scored popular science and personal websites the highest, with an average score of 5.94; the second highest average score, 5.21, was for websites of public welfare organizations; and the third highest average score, 4.25, was for websites of universities, research institutes, hospitals, and governments. Volunteers gave the lowest score to corporation websites and advertisement websites, with an average of only 3.80. By interviewing and communicating with volunteers, we found that they had various Internet search habits. In most cases, users may go through the first few pages of the search results of a particular search engine. When the results are not good enough, they change the keywords to search again. They often choose additional keywords suggested by the search engines when they redo the search. Most users check only the search results listed on the first summary page. Some use different search engines for the same keyword search. Users who have little search experience often blindly trust the results from the search engines. They often believe that the top-ranked result is the best one. During the interviews, users also gave us some good suggestions for collecting data. For example, we provided them with standard websites, but did not provide them with a standard for low scores. Although the standards were authoritative, they could not accommodate various needs of different users; without a medical background, users may have a hard time evaluating professional websites.

Using search engines to obtain health and medical information is an effective method for most Internet users. Our study indicates that the four major search engines, Google, Yahoo!, Bing, and Ask.com, are all helpful to users in their health and medical searches. Thus, they are used and recommended by most consumers for obtaining medical information online. However, there is significant room for improvement, especially in getting more relevant and comprehensive information, as well as in ranking the websites according to their usefulness. In this regard, there is no gold standard, and the various search engines each have their own merit, although Google and Bing are more advanced than others. Furthermore, the various search engines have different focuses on their search content. Hence, we suggest that users apply multiple search engines when looking for medical and health information online, instead of using only a single search engine.

Our study complements some earlier studies in evaluating Internet searching for health and medical information. Many of the previous studies emphasized the quality or reliability of the content [33]. In contrast, our study has a new focus—that is, usability—which more directly reflects the perspectives of actual users than most previous work. Our method is also significantly different from earlier approaches. For example, a previous usability study [34] was carried out in a usability laboratory. While conducting a study in a usability laboratory has some advantages, it also has some limitations. In particular, a usability laboratory is an artificial environment, and some participants may have different usage behavior especially under the time pressure of a study, as Eysenbach and Köhler [34] reported. Therefore, we used another method of usability testing: hallway testing. To best ensure that each participant sufficiently evaluated each webpage of the sample search results, we did not give a deadline for the evaluation and we only advised each participant to finish the evaluation as soon as possible to reduce the chances of possible changes and expirations of the websites (with the actual timeframes of the study, we believe such chances are negligible for any conclusion drawn in this paper). Some of our conclusions are in agreement with Eysenbach and Köhler [34]. For example, both studies found that consumers find information from popular science and personal websites and the websites of public welfare organizations acceptable. Nevertheless, our study sheds some new light on the usability of the various Internet search engines. Overall, our study adds significant value to the understanding of Internet searching for health and medical information.

In summary, our study may provide a useful analysis of medical information Internet searching and some helpful suggestions for improving the overall usability of health-related Internet searches. It provides some information to Internet users in terms of whether to use multiple search engines and how to use them. It also gives some informative data for Internet search engine developers to improve their search engine or to develop a medicine-specific search engine.

Our study also has some limitations. It had a limited sample size and used only one search keyword. Furthermore, the volunteers’ backgrounds were relatively homogeneous, as they were all highly educated, usually in science. More large-scale studies with participants of diverse backgrounds are needed for conclusive results. While this study provides Internet users with an informative reference and some guidance for obtaining medical information online, we will conduct a much larger-scale study with more representative samples in the future. In particular, we will include cancer patients and their family members in the study.

## Multimedia Appendix 1

Summary of the data and analyses. RN: rank of each result in the search list. The RN starts with 1001 in blue for Google results, 2001 in pink for Yahoo!, 3001 in green for Bing, and 4001 in red for Ask.com. Website name: the title of search result website. Website URL: the URL for search result website. GPR: Google PageRank Value (N means unavailable). Type: the four content types of search result websites. V1 to V8: the eight volunteers’ scores of search results. AVG: the average score of a search result from all 8 volunteers. G, Y, B, A: the RN of the same website in the search results of Google, Yahoo!, Bing, and Ask.com, respectively. Main_Breast Cancer: frequency of keyword “Breast Cancer” in main page of search result. Sub_Breast Cancer: frequency of keyword “Breast Cancer” in main and subpage of search results combined.

## Abbreviations

SNOMED CT: Systematized Nomenclature of Medicine-Clinical Terms
